# Does concealing familiarity evoke other processes than concealing untrustworthiness? – Different forms of concealed information modulate P3 effects

**DOI:** 10.1017/pen.2019.4

**Published:** 2019-07-23

**Authors:** René Koeckritz, André Beauducel, Johanna Hundhausen, Anika Redolfi, Anja Leue

**Affiliations:** 1 Institute of Psychology, University of Kiel, Kiel, Germany; 2 Institute of Psychology, University of Bonn, Bonn, Germany

**Keywords:** concealed information, P3 amplitude, concealed information test, trustworthiness, injustice sensitivity

## Abstract

It was investigated whether concealing learned stimulus attributes (i.e., trustworthiness vs. untrustworthiness) has similar effects on the P3 amplitude than concealing stimulus familiarity. According to salience hypothesis, known, deceptive stimuli (probe) are (perceived) more relevant than truthful, unknown stimuli (irrelevant) evoking a more positive probe P3 amplitude. When all stimuli are known, concealing information is more cognitively demanding than non-concealing information evoking a less positive P3 amplitude according to the mental effort account. Ninety-seven participants concealed knowledge of previously learned faces in the familiarity condition (probe vs. irrelevant stimuli). In the trustworthiness condition, participants concealed untrustworthiness to previously learned faces and responded truthfully to previously learned trustworthy and untrustworthy faces (known, concealed vs. known, truthful stimuli). The parietal mean P3 amplitude was more positive for probe stimuli than for irrelevant stimuli in the familiarity condition providing evidence for the salience hypothesis. In the trustworthiness condition, concealing untrustworthiness showed the smallest parietal mean P3 amplitude suggesting evidence for the mental effort hypothesis. Individual differences of perpetrator’s sensitivity to injustice modulated the P3 amplitude in the trustworthiness condition.

Concealing information is a behavior that occurs in different legal and social situations (e.g., in social interactions). Especially in legal psychology, there is a high interest in developing methods to differentiate between concealed and non-concealed physiological patterns. The differentiation between truthful and non-truthful responses proves to be difficult because behavioral indicators like gestures, facial expression, and intonation are not very reliable (e.g., DePaulo et al., 2003). In this respect, techniques like the electroencephalography (EEG) and functional magnetic resonance imaging (fMRI) constitute promising neuroscientific approaches to investigate concealed versus non-concealed information (e.g., Gamer, Bauermann, Stoeter, & Vossel, 2007; Meijer, klein Selle, Elber, & Ben-Shakhar, 2014). An event-related potential (ERP) that gains a lot of attention in the field of deception is the P3 component. The P3 component typically occurs between 300 and 800 ms post-stimulus (Donchin & Coles, 1988; Fabiani, Gratton, Karis, & Donchin, 1987; Johnson, 1993) and is often regarded as an indicator of stimulus salience (e.g., Kok, 2001; Leue & Beauducel, 2015; Meijer et al., 2014; Verschuere, Ben-Shakhar, & Meijer, 2011) in the context of concealed information tests (CIT). Some CIT studies reported earlier time windows for the P3 amplitude starting between 200 and 280 ms post-stimulus (Hahm et al., 2009; Jung, Kang, & Kim, 2013; Leue, Lange, & Beauducel, 2012). It has been found that concealed familiar stimuli in CITs lead to larger P3 amplitudes than unfamiliar stimuli. In CITs, familiar stimuli, whether concealed or not, induce larger P3 amplitudes than unfamiliar stimuli. The larger P3 amplitudes induced by known or familiar stimuli have been related to the recognition of stimulus salience. It is, however, likely that the recognition of salient stimuli is not the only relevant process involved in CIT. Besides the recognition of stimulus salience, the orienting response and arousal inhibition have been discussed as relevant processes (klein Selle, Verschuere, Kindt, Meijer, & Ben-Shakhar, 2015; Rosenfeld, Ozsan, & Ward, 2017). New versions of CIT protocols have been developed that allow for an improved identification of arousal-related processes that may occur beyond the orienting response (klein Selle et al., 2015; Rosenfeld et al., 2017). We also assume that processes beyond the recognition of familiar (salient) stimuli are involved in CIT. Our focus is more on the differentiation between cognitive processes following stimuli that are associated with deceptive versus truthful responses. Following Johnson (2014), we assume that stimuli requiring deceptive responses share similarities with dual-task paradigms because known information is suppressed and deceptively responded to. Accordingly, we assume that the processing of known stimuli associated with deceptive responses requires more mental effort than the processing of known stimuli associated with truthful responses.

Results on individual differences in CITs suggest that processes related to concealing information affect the P3 amplitude in addition to stimulus salience (Leue & Beauducel, 2015; Leue et al., 2012). Cognitive processes related to the inhibition or suppression of deceptive information may also be relevant in CITs. These cognitive processes may require additional resources (Johnson, 2014). Johnson (2014) considered deceptive responding in a dual-task context, where one task is the identification of the truthful response and the second task is the deceptive response. Accordingly, we expect that suppressing knowledge of known information requires additional cognitive resources because a second task (suppression) adds on the initial task (knowledge of information). To investigate the mental effort hypothesis, we modified the CIT protocol in a way that cognitive processes related to the deceptive responses (e.g., suppression of knowledge and responses) become more relevant than cognitive processes related to the discrimination of familiar and unfamiliar stimuli. Till date it has not yet been investigated whether concealing stimulus attributes (like trustworthiness or negative evaluations of others) of known stimuli has effects on the P3 amplitude. Moreover, the P3 amplitude has been shown to be sensitive to several aspects of the experimental setting. For example, in some paradigms the P3 component has been regarded as a reversed indicator of working-memory load (Mecklinger, Kramer, & Strayer, 1992; Zhou & Thomas, 2015) and mental effort (Beauducel, Brocke, & Leue, 2006; Kok, 2001). It is therefore not clear whether concealing or not concealing stimulus attributes of known stimuli affects the P3 amplitude. Therefore, the present study compares the P3 amplitudes of a CIT with the P3 amplitudes of an adapted CIT that occur when stimulus attributes of known stimuli are concealed or not.

## CITs and the salience hypothesis

The CIT (originally named as Guilty Knowledge Test, GKT, Lykken, 1959) has been frequently applied to differentiate concealed versus non-concealed information by means of the parietal P3 amplitude (e.g., Ambach, Bursch, Stark, & Vaitl, 2010; Farwell & Donchin, 1991; Gamer & Berti, 2010; Meixner & Rosenfeld, 2011; Rosenfeld, Hu, & Pederson, 2012). Most CITs comprise three different kinds of stimuli: (1) probe, (2) target, and (3) irrelevant stimuli. Probe stimuli are known to participants, and they are requested to conceal their knowledge of these stimuli. Target stimuli are also known to participants, and participants are asked to respond truthfully to these stimuli. Irrelevant stimuli are unknown stimuli and require a truthful response. CITs differ with respect to the stimulus content by presenting objects like bracelets or social stimuli like faces participants should respond to deceptively or truthfully.

A considerable number of social and legal CIT-like studies have shown larger parietal P3 amplitudes for probes than for irrelevant stimuli indicating that probes are perceived more salient than irrelevant stimuli supporting the salience hypothesis (e.g., Gamer & Ambach, 2014; Gamer & Berti, 2010; Leue & Beauducel, 2015; Leue et al., 2012; Rosenfeld, Miller, Rao, & Soskins, 1998). As probe stimuli are known to participants whereas irrelevant stimuli are in some studies unknown prior to task performance or not relevant for deception, the effect of perceived stimulus salience on the P3 component arises from a difference between known stimuli including concealing knowledge and unknown stimuli associated with truthful responses (named as familiarity effect in this study). Since it is likely that the P3 amplitude differences between probe and target stimuli on the one hand and irrelevant stimuli on the other hand are primarily due to the differences in stimulus familiarity in CIT, it remains to be investigated whether and how concealing information can be represented by the P3 amplitude when the CIT is modified. The effect of familiarity on P3 amplitudes in a CIT can also be related to the affective stimulus salience. Kayser, Bruder, Tenke, Stewart, and Quitkin (2000) suggested that especially the early P3 component can be regarded as an indicator of initial affective stimulus salience. As both the early P3 and late P3 components have a parietal topography, the early P3 component might not necessarily be regarded as a traditional P3a or as a novelty P3, which typically has a frontal topography (Leue & Beauducel, 2015; Polich, 2007). Barry, Steiner, and De Blasio (2016) show evidence for a differentiation of the P3a, P3b, and the novelty P3.

## Concealed information and the mental effort hypothesis

The identification of concealed information or active lying is not only relevant in legal settings but also in business settings (e.g., Lindsey, Dunbar, & Russell, 2011; Strout, 2002). To investigate the generalizability of early and late P3 effects found in CITs for concealed familiarity compared to concealed stimulus attributes we instructed participants to conceal untrustworthiness. Trustworthiness is important for the quality of social interactions in business fields. Everyday business involves many different social interactions, and in this respect interaction partners may show different levels of trustworthiness. Studies show that trustworthiness represents a highly valued personal characteristic (Anderson, 1965; Schönbach, 1972) and people show a higher tendency to engage in interactions with others who seem to be trustworthy (Yang, Qi, Ding, & Song, 2011). Therefore, it can be expected that people may regard it as unpleasing to describe a colleague as trustworthy, who in fact is not evaluated as trustworthy. Therefore, concealing untrustworthiness (i.e., saying a person is conceived to be trustworthy although (s)he is in fact not conceived as trustworthy) should be more cognitively demanding than truthful evaluations of trustworthiness.

To transfer the traditional CIT to a business context the trustworthiness of colleagues had to be denied. We used faces as stimuli in the trustworthiness condition and in the familiarity condition because they are of special relevance for social interactions. The social relevance of faces may enhance the effects of recognition of salient stimuli as well as the effects of mental effort while untrustworthiness is concealed. The new *trustworthiness-condition* was realized with three stimulus categories: (1) faces predefined as untrustworthy required a deceptive response because participants indicated by their button response that they would evaluate the faces to be trustworthy faces (concealed untrustworthiness, which is comparable to probe stimuli in a traditional CIT, therefore for short: untrustworthiness-probe), (2) faces predefined as untrustworthy required a truthful response by button press (untrustworthy stimuli), and (3) faces predefined as trustworthy required a truthful response by button press (trustworthy stimuli). Thus, the only expected difference in processing the untrustworthiness-probe and the (un)trustworthy stimuli is that the latter require a truthful response whereas the untrustworthiness-probe requires a concealed response to the stimulus attribute. It should be noted that in the trustworthiness condition, a classical irrelevant category of unknown stimuli was not available. This might be due to the fact that an evaluation of the trustworthiness of completely unknown individuals would probably reflect emotional expressions of the faces (Todorov, Pakrashi, & Oosterhof, 2009) or social stereotypes (Sutherland, Young, Mootz, & Oldmeadow, 2015). Consequently, in the trustworthiness condition all three types of stimuli were known to participants prior to task performance. Therefore, concealing untrustworthiness requires the processing of known stimuli and in addition the preparation of a deceptive response. If concealing untrustworthiness would be indeed a supplementary cognitive process that requires mental resources (e.g., for suppressing a relevant social evaluation), one might expect that another cognitive hypothesis might better account for the explanation of P3 differences than the salience hypothesis. A promising alternative account to the salience hypothesis would be the mental effort hypothesis (Beauducel et al., 2006; Kok, 2001). The mental effort hypothesis presumes that overlapping processes leave reduced cognitive capacity available for stimulus processing. That is why the P3 amplitude to untrustworthiness-probe might be less positive than the P3 amplitude to known stimuli (i.e., predefined untrustworthy faces, predefined trustworthy faces). This expectation is in line with Johnson (2014), who assumes that deceptive responding can be regarded as a form of dual-task where the amount of processing required for inhibiting truthful responses reduces the capacity for deceptive responding. Although Johnson (2014) discusses these effects in relation to a reduction of the late positive potential (LPP), his results match the reduction of the P3 that has been found when additional resources are required so that they are unavailable for stimulus processing itself (Beauducel et al., 2006; Mecklinger et al., 1992). Moreover, the LPP is enhanced by the affective valence of stimuli (Baum, Rabovski, Rose, & Rahman, 2018) showing that the LPP is similar to the P3 also with respect to stimulus salience.

## Individual differences in CITs

To further elucidate the meaning of the neural processes of the P3 amplitude while people conceal knowledge, Leue et al. (2012) recommended taking the moderating role of individual differences into account. Leue et al. (2012) showed that a scale for the measurement of the sensitivity of the behavioral inhibition system (BIS), which is termed trait-BIS (i.e., individual differences of aversiveness and conflict sensitivity, Carver & White, 1994), and perpetrator’s sensitivity to injustice (SI-perpetrator, Schmitt, Baumert, Gollwitzer, & Maes, 2010; Schmitt, Neumann, & Montada, 1995) modulate the P3 effect during deception. Higher trait-BIS and higher SI-perpetrator scores were related to a more pronounced increase of the early P3 amplitudes from irrelevant to probe pictures. The authors suggest that concealing knowledge leads to an intensification of stimulus salience for individuals with higher trait-BIS and higher SI-perpetrator scores. Leue and Beauducel (2015) demonstrated that women with higher SI-perpetrator scores demonstrated larger early P3 differences between probe and irrelevant stimuli whereas men did not show this effect. These findings indicate that women and men may have different ways to process ethically salient information (Dalton & Ortegren, 2011; Donoho, Heinze, & Kondo, 2012). Unethical responses seem to be more salient to women because they recognize more intensely that they behave against social rules, whereas men tend to process ethically salient information with a justice orientation (e.g., unethical responses are justified because of task requirements). Therefore, the effect of sex on the P3 amplitude will also be explored. As an extension of prior findings on individual differences (Leue & Beauducel, 2015; Leue et al., 2012) we expected that the cognitive processes underlying concealed untrustworthiness are more intense for individuals with higher trait-BIS and higher SI-perpetrator scores.

## Aims and hypotheses

In sum, this study investigated individual differences and cognitive processes of concealed information by means of the P3 amplitude in two task conditions. A more comprehensive understanding of the cognitive processes and individual differences involved in a CIT may help to further improve this assessment tool for business and forensic contexts. Moreover, the identification of cognitive processes beyond the recognition of stimulus salience may further our theoretical understanding of deception. Since a conventional CIT serves to investigate the P3 amplitude difference between concealed familiarity of stimuli and truly unknown stimuli, the CIT is a sophisticated tool for the identification of concealed familiarity of stimuli. In one condition of the present study that was close to the conventional CIT, we investigated effects of stimulus salience that asked participants to conceal knowledge to known faces and to respond truthfully to other known versus unknown faces (subsequently named as familiarity condition). This familiarity condition tests the recognition of salience hypothesis presuming that the probe P3 is larger than the irrelevant P3 (hypothesis a). The recognition of familiar/known stimuli determines the P3 amplitude difference for the known, probes versus the unknown, irrelevants in a conventional CIT.

When all stimuli are known, another cognitive process beyond recognition of salient stimuli is presumed to determine the P3 difference between truthful responses and deceptive responses – mental effort. To investigate this process, we provided a second condition in that participants had to conceal the untrustworthiness of known faces. With regard to the trustworthiness condition, we expected that concealing untrustworthiness costs more mental effort (smaller P3 amplitude) than the processing of trustworthy or untrustworthy stimuli associated with truthful responses (hypothesis b). Individuals with higher trait-BIS scores and higher SI-perpetrator scores, respectively, should reveal more perceived stimulus salience (larger P3 amplitudes) in the familiarity condition compared to individuals with lower trait-BIS and SI-perpetrator scores (hypothesis c). If mental effort effects account for concealing untrustworthiness, the untrustworthiness-probe P3 amplitude should be smaller in individuals with higher trait-BIS and SI-perpetrator scores, respectively (hypothesis d). Effects of sex on the P3 amplitude will be explored.

## 1 Method

### 1.1 Participants

A total of *N* = 104 individuals voluntarily participated in the study. Participants (*n* = 7) with an insufficient number of trials per picture type (i.e., less than 20 trials per picture type) were excluded from the present study. Exclusion of participants was due to artifacts that could not be corrected by means of Independent Component Analysis (ICA). Thus, *N* = 97 (49 male, age: *M* = 24.58 years, SD = 4.70 years, range = 18–38 years) remained for statistical analysis. Sixty-one students and five employees were recruited at the University of Kiel. Thirty students and one employee were recruited at the University of Bonn. There were no significant differences in age, trait-BIS scores, SI-perpetrator scores, and gender distribution between the subsamples from the two universities (all *p*s > .27; see Table 1). Handedness (i.e., the preference to perform a variety of tasks with the one or the other hand) was measured by means of the Edinburgh Handedness Inventory (EHI) with 10 items (Milenkovic & Dragowic, 2013; Oldfield, 1971). All included participants were right-handed (EHI score: *M* = 89.51, SD = 14.28) and had a normal or corrected-to-normal vision. Participants obtained a reimbursement of €10, or they received two credit hours for taking part in this study. Additionally, every participant could win a maximum of €5 for correct responses in the deception task (see below). The authors realized the protocol of the ethical standards as in former EEG studies on concealed information, which were approved by the local ethical committee. The study was carried out in accordance with the recommendations of the Helsinki (2013) declaration with written informed consent from all subjects. The protocol of a former study (Leue et al., 2012, *Frontiers in Psychology*) that has been extended in this study was approved by the Ethics Committee of the German Psychological Society while scientific experts reviewed the research proposal that the second and the last authors had submitted to the German Research Foundation.

**Table 1.** Descriptive statistics for the subsamples from two universities

Measure	University of Kiel (*N* = 66)	University of Bonn (*N* = 31)	Significance of difference
Age	23.81 (4.62)	24.94 (4.73)	.27
Trait-BIS	2.93 (0.64)	3.04 (0.55)	.36
SI-perpetrator	3.67 (0.79)	3.69 (0.74)	.90
Gender distribution	35 females, 31 males	13 females, 18 males	.31

*Notes*: The standard deviation is given in parentheses. Independent samples t-tests were performed for Age, Trait-BIS score, and SI-perpetrator score, and a χ²-test was performed for the difference of gender distributions.

### 1.2 Measures

Participants filled in the German version of the BIS/BAS scales (Strobel, Beauducel, Debener, & Brocke, 2001). The BIS/BAS scales measure an individual’s sensitivity to aversiveness (trait-BIS) and an individual’s sensitivity to appetitive reinforcement (trait-BAS) with 24-items using a 4-point Likert-type answer format (1 = applies exactly; 4 = applies not). We focused on seven items comprising the trait-BIS scale to investigate individual differences of the P3 (Cronbach’s α: 0.85). Furthermore, participants filled in the SI-questionnaire (Schmitt et al., 2010) measuring individual differences in SI for different perspectives (perpetrator, victim, observer, beneficiary). The SI-questionnaire contains 40 items with a six-point answer format (0 = not at all; 5 = strong agreement). In accordance with previous P3 studies on individual differences in deception (Leue & Beauducel, 2015; Leue et al., 2012), we focused our analysis on the SI-perpetrator subscale (10 items; Cronbach’s α: 0.70). After finishing the deception task, participants evaluated their general motivation for taking part in this experiment and the motivation to conceal their knowledge. Both evaluations were rated on a 9-point Likert scale (ranging from 1 = not motivated to 9 = highly motivated). In our sample of *N* = 97 participants the mean was 7.42 (SD = 1.85) for instruction-conform performance of the deception task. That is participants were (in accordance with the range of answer categories) highly motivated to perform the task conditions not simply as a stimulus-response assignment but related to the context.

### 1.3 Deception task and experimental design

The experimental task comprised two conditions (1) concealing familiarity of faces (for short: familiarity condition) and (2) concealing trustworthiness of faces (for short: trustworthiness condition). The familiarity condition was similarly designed to Leue et al. (2012): Participants were asked to conceal their knowledge to *familiar-probe* pictures by pressing on the right cursor button. They also should press the right cursor button following *irrelevant* pictures to indicate truthfully that irrelevant pictures were completely unknown. Participants should press the left cursor button to indicate truthfully that they knew the target pictures. Correct responses to the pictures resulted in a win feedback (+2Ct) and incorrect responses (probe and irrelevant: left cursor button; target: right cursor button) resulted in a loss feedback (−2Ct). If participants did not react within the 2000 ms response interval, they received a loss feedback (−2Ct). Four different probe pictures and four different target pictures were selected, whereas 19 irrelevant pictures were chosen (4:4:19 ratio, for details on pictures see Supplement S1). All probe and target pictures were presented 10 times. Each irrelevant picture was presented twice, whereas two of the irrelevant pictures were presented three times. Altogether participants performed 120 trials (40 probe, 40 irrelevant, and 40 target items) presented in 10 blocks with 12 pictures per block in a pseudo-random order. It is noteworthy that we differentiate between the picture-type ratio and the total number of repeated presentations for each picture type. The picture-type ratio for probe, target, and irrelevant of about 1:1:4 corresponds to prior 3-stimulus CITs, whereas the number of repeated presentations of each picture type is equal for each picture type (Cutmore, Djakovic, Kebbell, & Shum, 2009; Gamer & Berti, 2010; Jung et al., 2013; Kubo & Nittono, 2009; Meijer, Smulders, Merckelbach, & Wolf, 2007).

In the trustworthiness condition, participants were instructed to conceal their instructed attitude on untrustworthiness. That is, faces shown on the *untrustworthy-probe* pictures were predefined by the experimenter to be untrustworthy. Participants were asked to indicate by right cursor button presses that the face on untrustworthy pictures is trustworthy (untrustworthy-probe). They should press the left cursor button on *untrustworthy* pictures to indicate truthfully that the face on the pictures (informed by instruction, see Supplement S2) was not trustworthy. On *trustworthy* pictures, participants had to respond with the right cursor button to indicate truthfully that the face on the pictures (informed by instruction) was trustworthy. Trustworthy pictures in the trustworthiness condition were identical in response to target pictures in the familiarity condition. The feedback to correct and incorrect responses of the trustworthiness condition was equivalent to the familiarity condition. Four different untrustworthy-probes, four different trustworthy pictures, and four different untrustworthy pictures were selected (4:4:4 ratio). Each picture was presented 10 times. Altogether participants performed 120 trials (40 probe, 40 trustworthy, and 40 untrustworthy items) presented in 10 blocks with 12 pictures per block in a pseudo-random order. Participants performed the second condition after a 2-min break. To control for effects of task sequence, half of the participants were randomized to the task sequence familiarity condition – trustworthiness condition (*n* = 48) and the other half was randomized to the task sequence trustworthiness condition – familiarity condition (*n* = 49).

**Figure 1.**
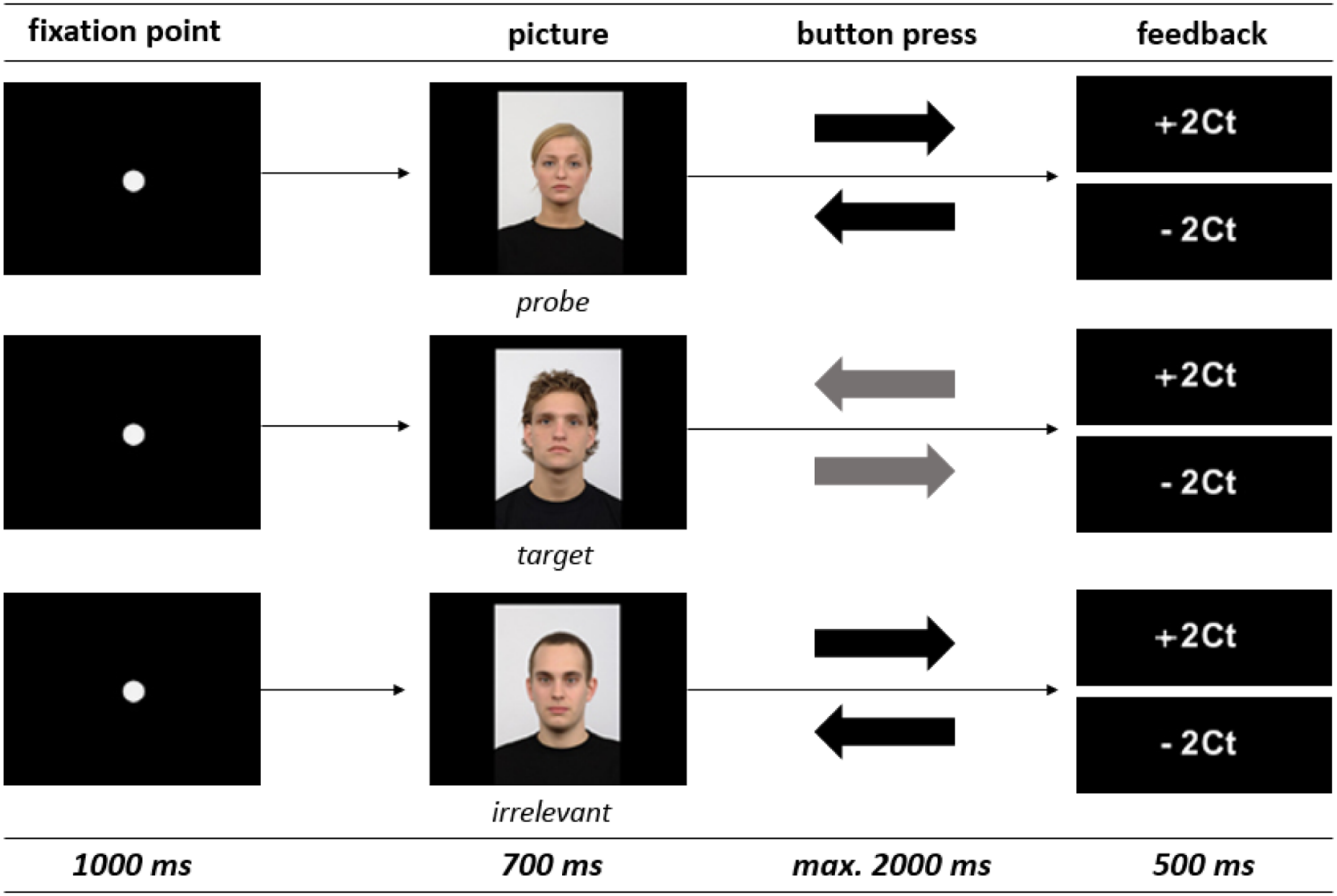
Trial sequence of a probe item, a target item, and an irrelevant item. The inter-trial-interval (ITI), which was 1000, 1500, or 2000 ms, is not presented in the figure. The trial sequence of the familiarity was equivalent to trustworthiness condition.

Each trial in the practice and the main task phase (Figure 1) consisted of a fixation point that was presented in the center of the 17˝ TFT screen for 1000 ms followed by a picture presented for 700 ms (picture size: 327 × 479 pixel; 96 dpi). Participants were instructed to respond to the left or right cursor button depending on picture type as fast and as accurately as possible. When the picture disappeared after 700 ms, participants could respond up to a maximum of 2000 ms while the screen remained black. After each response interval the feedback (+2Ct for correct response or −2Ct for incorrect response) was displayed for 500 ms. The inter-trial-interval (ITI) varied in a pseudo-random order between 1000, 1500, and 2000 ms. During the ITI the screen remained black. The trial sequence was identical to the study of Leue et al. (2012), but there were also some differences regarding picture material, number of pictures, response button, and feedback (Table 2).

**Table 2.** Comparison of the experimental designs

	Leue et al. (2012)	Present study
Picture material	International Affective Picture System (Bradley & Lang, 2007) All picture types were comparable regarding valence and arousal Pictures showed individuals from different perspectives with different clothing	Radboud faces database (Langner et al., 2010) All picture types were comparable regarding intensity, authenticity, and valence Pictures showed faces from frontal perspective wearing consistent clothing
Picture types (quantity)	Probe (3), target (3), irrelevant (20)	*Familiarity condition:* probe (4), target (4), irrelevant (19) *Trustworthiness condition:* *untrustworthiness-*probe (4), trustworthy (4), untrustworthy (4)
Number of trials per picture type	50 probe, 50 target, 50 irrelevant (overall 150 trials)	*Familiarity condition:* 40 probe, 40 target, 40 irrelevant (overall 120 trials) *Trustworthiness condition:* 40 untrustworthiness-probe, 40 untrustworthy, 40 trustworthy (overall 120 trials)
Response (button press)	Probe (left), irrelevant left), target (right)	*Familiarity condition:* probe (right), target (left), irrelevant (right) *Trustworthiness condition:* untrustworthiness-probe (right), untrustworthy (left), trustworthy (right)
Response feedback	Correct response: +5Ct False response: −15Ct No response: black screen False feedback in case of correct response: −15Ct (5 out of 20 irrelevant pictures)	Correct response: +2Ct False response: −2Ct No response: −2Ct False feedback in case of correct response: none

*Note*: f = familiarity condition, t = trustworthiness condition. Correct response means in accordance with task instruction. False response means that button press was not in accordance with task instruction. Explanation of the differences to Leue et al. (2012): We aimed at keeping the stimulus material constant and comparable in face expression and presentation. This has been realized in the Radboud faces database. To ensure that P3 variations are not due to variations of the number of pictures per picture type we used the reported and identical number of picture types per task condition. Monetary feedback varied in a range of our prior studies on reinforcement-related ERP tasks.

### 1.4 Procedure

After arriving, participants gave written informed consent and filled in the EHI. The experimenter explored the actual physical condition (e.g., duration of sleep the day before examination) and demographic variables (e.g., age, gender, education). Participants suffering from a neurological disease reporting unusual alcohol consumption, medical, or drug use were excluded, because these aspects can bias the P3 amplitude (Picton, 1992). After interviewing, participants were prepared for EEG recording. Both task conditions and the task-related instructions were presented with Presentation V18.1 (Neurobehavioral Systems, Albany, USA). Participants sat in an upright position so that they could comfortably see the instructions and items on the 17˝ TFT screen.

In the initial phase of the familiarity condition, participants read the task instruction. Then participants learned the four probe pictures and the four target pictures for 5 min, whereas irrelevant (unfamiliar) pictures were not learned. Before task performance, participants again read the instructions. Afterward, participants learned the eight faces again for 1 min and performed eight practice trials (four familiar-probe pictures, four target pictures). Probe and target faces of the familiarity condition were presented on the same screen with the four probe faces presented in the first line and the four target faces presented in the second line.

For the trustworthiness condition, a social context was set up because trustworthiness (in contrast to evaluations of familiar versus unfamiliar faces in the familiarity condition) is a social phenomenon that can be more convincingly activated in participants by means of a vignette (for the relevance to generalize from brain to field see Kedia, Harris, Lelieveld, & van Dillen, 2017). A business context was chosen for the trustworthiness condition because in the business context external information on the (un-) trustworthiness of previously unknown people can be of special importance. The initial instruction of the trustworthiness condition set participants in the following context: In everyday life people work with different colleagues, who could be subjectively perceived to be more or less trustworthy. Following this description, participants learned four untrustworthiness-probe pictures, four trustworthy pictures, and four untrustworthy pictures for 7 min (see Section 1.3. description of the trustworthiness condition of the deception task). Participants were instructed to learn the 12 pictures of the trustworthiness condition within 7 min. Again, the four faces in the first line were entitled as probe pictures, the four faces in the second line were named as trustworthy pictures that require a truthful response, and the four faces in the third line were introduced as untrustworthy faces that also require truthful responses. The learning phase in the trustworthiness condition took 2 min more compared to the familiarity condition because participants had to learn three types of pictures instead of two types. Before task performance, participants were again instructed on the monitor. Afterward participants learned the 12 faces again for 1 min and performed 12 practice trials (four untrustworthy-probe, four trustworthy, four untrustworthy). All instructions are given in Supplement S2. The EEG was recorded in both task conditions. After completing the deception task participants filled in the BIS/BAS scales, the sensitivity to injustice questionnaire. Finally, they were paid depending on their task performance (max. €15), thanked, and debriefed.

### 1.5 EEG recording

**Figure 2.**
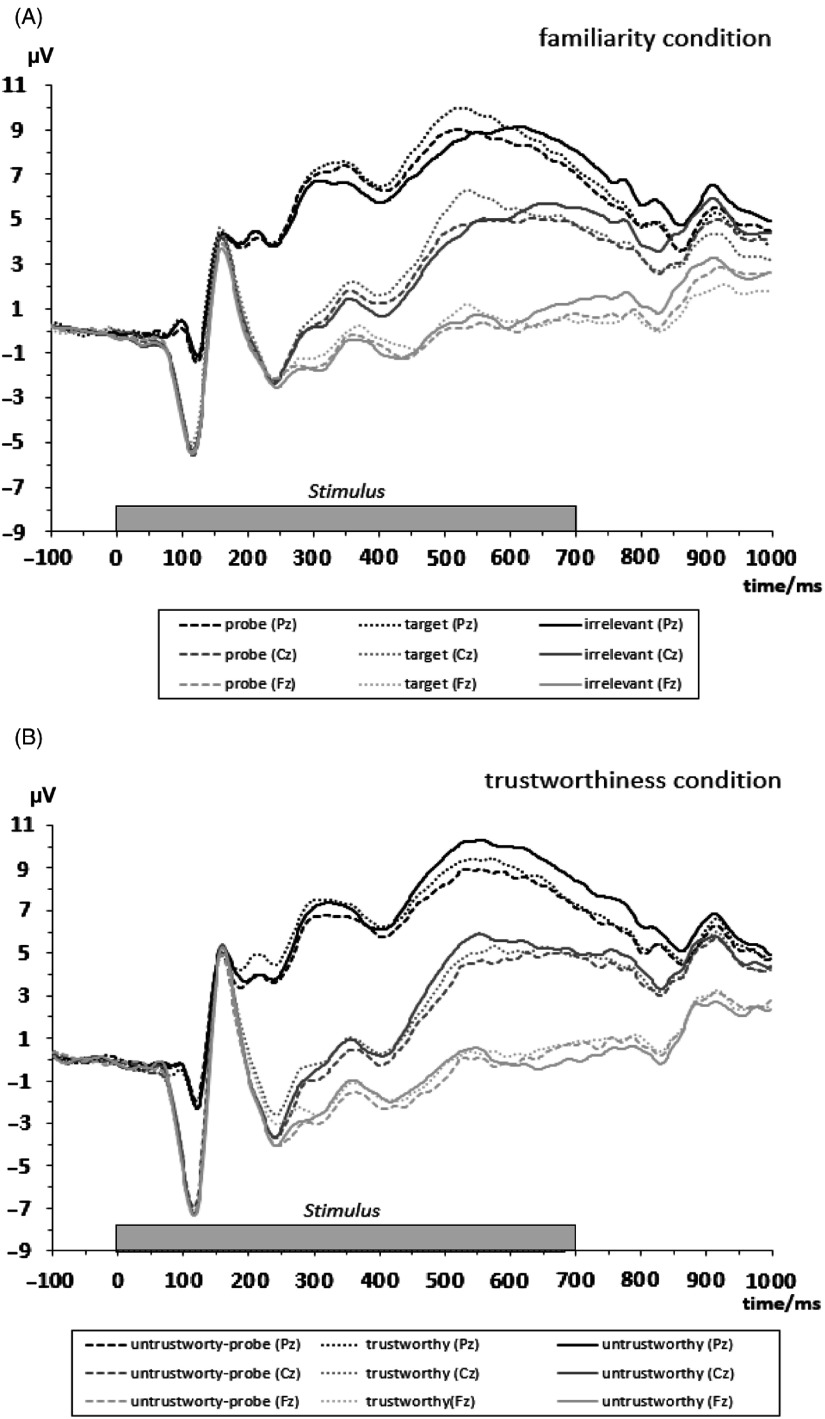
Stimulus-locked grand averages at Pz, Cz, and Fz separated for picture type of the familiarity condition (A) and of the trustworthiness condition (B). Dotted bar displays the stimulus presentation interval of 700 ms.

The EEG was recorded using active electrodes (Biosemi, Amsterdam, Netherlands) with 64 scalp active electrodes based on the extended 10/20 system (Jasper, 1958). The electrooculogram (EOG) was recorded from two horizontal electrodes placed beyond the epi canthi of both eyes and one vertical electrode located approximately 1 cm below the right eye. As per Biosemi’s design, the ground electrode during acquisition was formed by the Common Mode Sense active electrode and the Driven Right Leg passive electrode. All bioelectric signals were digitized on the laboratory computer system using ActiView software (Biosemi). Electrode offsets were kept below 30 mV during EEG recording. The EEG was sampled at 512 Hz. Offline analysis was performed by using EEGLab 13.5.4b based on MATLAB R2015a (The Math Works). All data were band-pass filtered (0.3–30 Hz) and were re-referenced to averaged mastoids. Independent Component Analysis (an automated infomax decomposition) was applied to correct for ocular artifacts. Further technical and muscle artifacts were rejected when the EEG signal exceeded ±85 µV. Artifact-free epochs with instruction-conform responses were separately segmented for the picture types. Participants included into statistical analysis had at least 22 artifact-free epochs for each picture type (familiarity condition: familiar-probe: *M* = 38.14, SD = 2.84; target: *M* = 38.07, SD = 3.03; irrelevant (unfamiliar): *M* = 38.08, SD = 3.11; trustworthiness condition: untrustworthy-probe: *M* = 37.89, SD = 3.20; untrustworthy: *M* = 37.76, SD = 3.66; trustworthy: *M* = 37.74, SD = 3.51). Grand averages of the picture-related ERPs (0–1000 ms, with 100 ms pre-stimulus-baseline) indicate an early P3 amplitude between 280 and 400 ms post-stimulus (Figure 2). The P3 component was quantified as a mean amplitude in the time interval between 280 and 400 ms post-stimulus. Since there is a second positive peak we added a supplement for an analysis of the mean amplitude in the time interval between 450 and 680 ms post-stimulus (Supplement S3) and a temporal principal component analysis of the ERP (Supplement S4).

### 1.6 Statistical analysis

For the dependent variables, percentage of correct responses and response times repeated-measures ANCOVAs were separately calculated for each task condition: The familiarity-ANCOVA comprised the repeated-measures factor Picture type (three levels: familiar-probe, target, irrelevant (familiarity condition) and Sex (level: male and female) as a between-subjects factor.

The trustworthiness-ANCOVA included the repeated-measures factor Picture type (three levels: untrustworthy-probe, trustworthy, untrustworthy) and Sex (levels: male and female) as a between-subjects factor.

Two separate repeated-measures ANCOVAs were performed for the mean P3 amplitudes. For the familiarity condition, Position (three levels: frontal (Fz), central (Cz), and parietal (Pz)) and Picture type (three levels: familiar-probe, target, irrelevant) were entered as repeated-measures factors. Sex (levels: male and female) was entered as a between-subjects factor. For the trustworthiness condition, Position (three levels: frontal (Fz), central (Cz), and parietal (Pz)) and Picture type (three levels: untrustworthy-probe, untrustworthy, trustworthy) were entered as repeated-measures factors. Sex (levels: male and female) was entered as a between-subject factor. Finally, an overall ANCOVA was performed with the aforementioned within-subject and between-subject factors as well as the within-subject factor Task condition (familiarity condition versus trustworthiness condition) and the between-subject factor Task sequence (familiarity-trustworthiness, trustworthiness-familiarity). In all repeated-measures ANCOVAs, the SI-perpetrator scale and Trait-BIS scale were entered as mean centered covariates. Violations of the sphericity assumption were corrected for all repeated-measures ANCOVA tests by means of Greenhouse-Geisser epsilon (ε), which is reported along with uncorrected degrees of freedom. Partial eta square *(*η*_p_^2^*) is reported to evaluate effect sizes. We checked for multiple testing and applied Bonferroni correction (i.e., significance level/*n*, with *n* as the number of tests of the same hypothesis) where necessary. Within the results section, the hypotheses were tested only once. Moreover, most of the findings also hold when we exclusively interpret results of simple contrast comparisons for stimulus type at an alpha level of *p* < .01. However, in the Supplement S3 we report additional results for the second positive P3 peak. Since the hypotheses were tested for a second time in the Supplement, we used a nominal alpha level of *p* < .025 for the additional significance tests.

## 2. Results

### 2.1 Behavioral data of the familiarity condition

A significant Picture type main effect was observed for the percentage of correct responses, *F*(2, 186) = 20.27, *p* < .01, *ε* =.99, *η_p_^2^* = 0.18. Simple contrasts revealed that the percentage of correct responses was significantly lower to familiar-probe compared to irrelevant pictures, *F*(1, 93) = 29.13, *p* < .01, *η_p_^2^* = 0.24, and to target compared to irrelevant pictures, *F*(1, 93) = 33.97, *p* < .01, *η_p_^2^* = 0.27 (Table 3). The percentage of correct responses to probe and target pictures did not significantly differ, *F*(1, 93) < 1, *p* = .96. There were no main effects of Sex, Trait-BIS, SI-perpetrator (all *p*s > .20) and also no interaction of Picture type × Sex and Picture type × Trait-BIS (all *p*s > .14) but a tendency of Picture type × SI-perpetrator, *F*(2, 186) = 2.89, *p* = .06, *η_p_^2^* = 0.03, for the percentage of correct responses to probe and target pictures.

**Table 3.** Percentage of correct responses (%) and mean response times (ms)

Familiarity			Trustworthiness		
*Picture type*	*Percentage of correct responses (%)*		*Picture type*	*Percentage of correct responses (%)*	
Familiar-probe	95.44	(0.64)	Untrustworthy-probe	97.58	(0.43)
Target	95.39	(0.75)	Untrustworthy	94.38	(0.91)
Irrelevant	98.95	(0.33)	Trustworthy	97.19	(0.51)
*Picture type*	*Mean response times*		*Picture type*	*Mean response times*	
Familiar-probe	954.08	(18.69)	Untrustworthy-probe	938.13	(21.27)
Target	972.94	(21.59)	Untrustworthy	931.04	(18.95)
Irrelevant	895.94	(16.34)	Trustworthy	937.41	(19.44)

Mean response times differed among Picture types, *F*(2, 186) = 21.31, *p* < .01, *ε* =.82, *η_p_^2^* = 0.19. Simple contrasts revealed that RT was significantly longer to familiar-probes compared to irrelevant pictures, *F*(1, 93) = 30.45, *p* < .01, *η_p_^2^* = 0.25 (Table 3). RT to target pictures was longer compared to irrelevant pictures, *F*(1, 93) = 26.56, *p* < .01, *η_p_^2^* = 0.22 (Table 3). RT was marginally significant longer to target compared to familiar-probe pictures, *F*(1, 93) = 2.89, *p* = .09, *η_p_^2^* = 0.03. There were no main effects of Sex, Trait-BIS, SI-perpetrator (all *p*s > .29) and also no interaction of Picture type × Sex, Picture type × Trait-BIS and Picture type × SI-p for mean response times (all *p*s > .12).

### 2.2 Behavioral data of the trustworthiness condition

A Picture type main effect was observed for the percentage of correct responses, *F*(2, 186) = 12.97, *p* < .01, *ε* = .74, *η_p_^2^* = 0.12. Simple contrasts revealed that the percentage of correct responses was significantly higher to untrustworthy-probe pictures compared to truthful untrustworthy pictures, *F*(1, 93) = 17.26, *p* < .01, *η_p_^2^* = 0.16 (Table 3). Moreover, the percentage of correct responses was significantly higher to truthful trustworthy pictures than to truthful untrustworthy pictures, *F*(1, 93) = 12.56, *p* < .01, *η_p_^2^* = 0.12. The percentage of correct responses to concealed untrustworthiness (trustworthiness-probe) and truthful trustworthy pictures did not significantly differ, *F*(1, 93) = 0.87, *p* = .35. There were no significant main effects of Sex, Trait-BIS, and SI-perpetrator (all *p*s > .58) and no interaction of Picture type × Sex, Picture type × Trait-BIS and Picture type × SI-perpetrator (all *p*s > .13). Mean response times did not differ across Picture types, *F*(2, 186) = 0.60, *p* = .54, *ε* =.93. There were no main effects of Sex, Trait-BIS, and SI-perpetrator (all *p*s > .10), no interactions of Picture type × Sex, and Picture type × Trait-BIS (all *p*s > .70).

**Figure 3.**
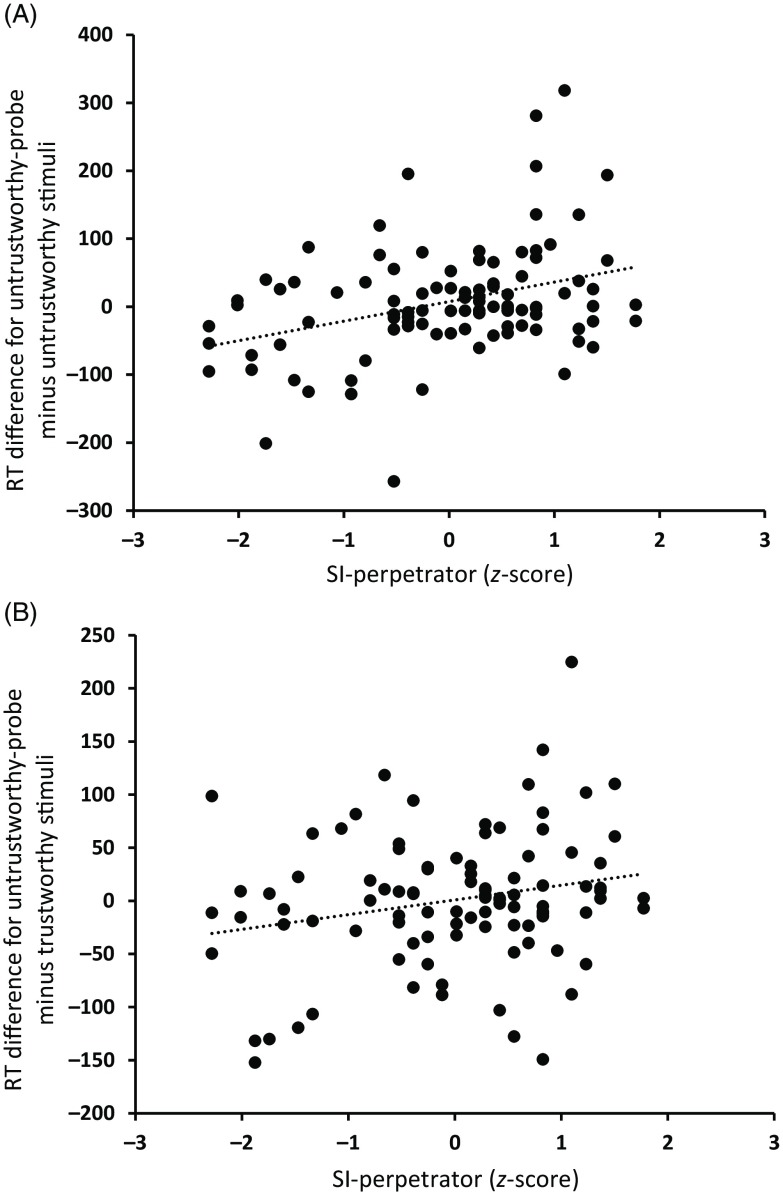
Scatterplot of the RT differences for untrustworthy-probe minus untrustworthy stimuli and the SI-perpetrator *z*-scores (A). Scatterplot of the RT differences for untrustworthy-probe minus trustworthy stimuli and the SI-perpetrator *z*-scores (B). RT is given in ms.

The interaction of Picture type × SI-perpetrator was significant for response times, *F*(2, 186) = 8.21, *p* < .01, *η_p_^2^* = 0.81. The RT difference between untrustworthiness-probes and untrustworthy pictures was higher for individuals with larger SI-perpetrator scores, as indicated by the positive Pearson correlation, *r*(97) = .35, *p* < .01, two-tailed (see Figure 3A). The RT difference between untrustworthiness-probes and trustworthy pictures was higher for individuals with larger SI-perpetrator scores, as indicated by the positive Pearson correlation, *r*(97) = .22, *p* < .05, two-tailed (see Figure 3B).

### 2.3 Mean P3 amplitude of the familiarity condition

For the P3 amplitude, there was a main effect of Position, *F*(2, 186) = 151.55, *ε* = .64, *p* < .01, *η_p_^2^* = 0.62. Simple contrasts revealed that the mean P3 amplitude was larger at Pz compared to Cz, *F*(1, 93) = 163.61, *p* < .01, *η_p_^2^* = 0.64, and it was larger at Pz compared to Fz, *F*(1, 93) = 167.78, *p* < .01, *η_p_* ^2^ = 0.64 (Table 4). Since the Position main effect indicated the typical parietal P3 topography, further analyses have been conducted at Pz. At Pz, the Picture type main effect was significant for the P3 amplitude, *F*(2, 186) = 7.05, *ε* = .96, *p* < .01, *η_p_^2^* = 0.07. Simple contrasts revealed that the P3 amplitude was larger for familiar-probes than for irrelevant stimuli, *F*(1, 93) = 6.41, *p* < .05, *η_p_^2^* = 0.06. There was no significant difference of the parietal P3 amplitude between probe and target stimuli, *F*(1, 93) = 1.27, *p* = .26. The P3a amplitude at Pz was significantly more positive (*M* = 7.23 µV, SE = 0.60) for targets compared to irrelevant pictures (*M* = 6.50 µV, SE = 0.54) in the familiarity condition, *F*(1,93) = 11.67, *p* < .01, *η_p_^2^* = 0.11. There was a marginal significant main effect of Sex, *F*(1, 93) = 2.98, *p* = .09, *η_p_^2^* = 0.31, with tendentially higher P3 amplitudes for females (*M* = 7.98 µV, SE = 0.84 µV) compared to males (*M* = 5.88 µV, SE = 0.83 µV). We did not find a P3 main effect of Trait-BIS or SI-perpetrator (all *p*s > .55). We also did not observe interactions of Picture type × Sex, Picture type × Trait-BIS, and Picture type × SI-perpetrator for the mean P3 amplitude (all *p*s > .14).

**Table 4.** Mean P3 amplitudes (in µV)

Familiarity			Trustworthiness		
*Electrode position*	P3 amplitudes		*Electrode position*	P3 amplitudes	
Pz	6.92	(0.57)	Pz	6.85	(0.52)
Cz	0.97	(0.53)	Cz	0.05	(0.57)
Fz	−0.90	(0.60)	Fz	−2.00	(0.59)
*Picture type* ^1^			*Picture type* ^1^		
Familiar-probe	7.04	(0.59)	Untrustworthy-probe	6.52	(0.51)
Target	7.23	(0.60)	Untrustworthy	6.90	(0.55)
Irrelevant	6.50	(0.54)	Trustworthy	7.14	(0.53)

*Note:* Standard error of mean is given in parentheses. ^1^Mean P3 amplitudes for each picture type at Pz.

### 2.4 Mean P3 amplitude of the trustworthiness condition

The main effect of Position was significant, *F*(2, 186) = 204.02, *ε* = .66, *p* < .01, *η_p_^2^* = 0.69. Simple contrasts revealed that the P3 amplitude was larger at Pz compared to Cz, *F*(1, 93) = 237.61, *p* < .01, *η_p_^2^* = 0.72, and it was larger at Pz compared to Fz, *F*(1, 93) = 224.19, *p* < .01, *η_p_* ^2^ = 0.71 (Table 4). Since the Position main effect indicated the typical parietal P3 topography, further analyses have been conducted for the P3 at Pz. At Pz, the Picture type main effect was significant, *F*(2, 186) = 5.21, *ε* = .99, *p* < .01, *η_p_^2^* = 0.05. Simple contrasts revealed that the P3 amplitude was significantly smaller for untrustworthiness-probe than for trustworthy stimuli, *F*(1, 93) = 10.93, *p* < .01, *η_p_^2^* = 0.11 (Table 4), and marginally significantly smaller for untrustworthiness-probe stimuli than for untrustworthy stimuli, *F*(1, 93) = 3.60, *p* = .06, *η_p_^2^* = 0.04. There were no main effects of Sex, Trait-BIS, or SI-perpetrator (all *p*s > .19) and no interactions of Picture type × Sex and Picture type × Trait-BIS (all *p*s > .18).

**Figure 4.**
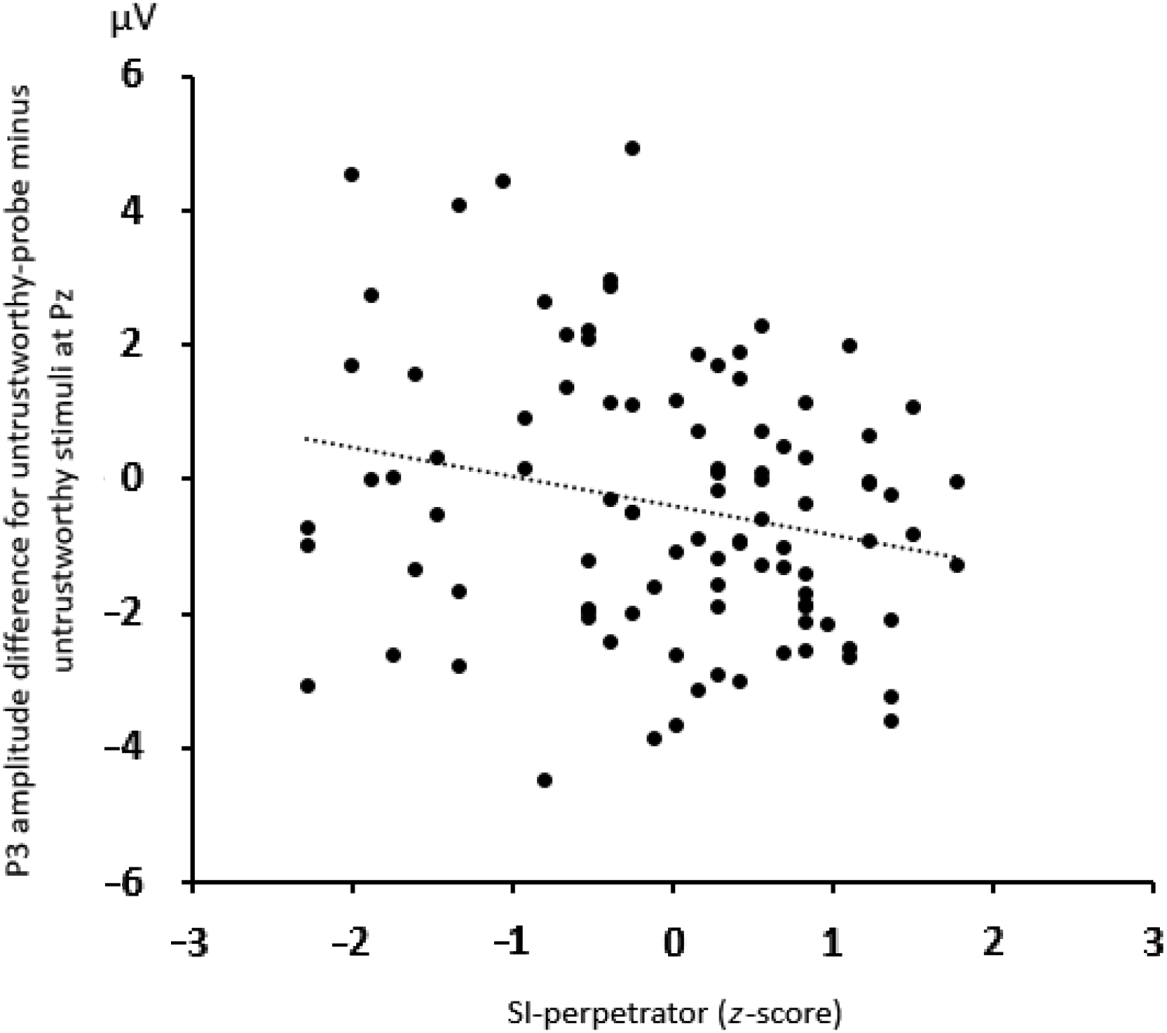
Scatterplot of the mean P3 amplitude differences for untrustworthy-probe minus untrustworthy stimuli and the SI-perpetrator *z*-scores. A high SI-perpetrator *z*-score means that the corresponding individual is in particular sensitive to injustice that she/he provides to others.

We found a marginally significant interaction of Picture type × SI-perpetrator, *F*(2, 186) = 2.55, *p* = .08, ε = .99, *η_p_^2^* = 0.03. The P3 difference score (untrustworthiness-probes minus untrustworthy pictures) was smaller for individuals with larger SI-perpetrator scores, as indicated by the negative Pearson correlation, *r(97)* = −.22, *p* < .05, two-tailed, see Figure 4. The difference between untrustworthiness-probes and trustworthy pictures did not significantly correlate with SI-perpetrator scores, *r(97)* = −.13, *p* = .22, two-tailed.

To investigate whether the Task condition had an effect on the results we investigated the early mean P3 amplitude in an overall repeated-measures ANCOVA including Position, both Task conditions (familiarity and trustworthiness), and Picture type as repeated-measures factors. Task sequence (familiarity-trustworthiness, trustworthiness-familiarity) was inserted as a between-subjects factor in addition to Sex. Covariates were again the mean-centered SI-perpetrator and Trait-BIS scales. The results were the same as reported earlier with two exceptions: The Position × Task condition interaction was significant for the early mean P3 amplitude, *F*(2, 184) = 7.07, *p* < .01, ε = .63, *η_p_^2^* = 0.07. The Task condition main effect tended to be significant at Cz, *F*(1, 92) = 3.78, *p* = .06, *η_p_^2^* = 0.04, with the mean P3 amplitude being less positive in the trustworthiness condition (*M* = 0.05 µV, SE = 0.57) and more positive in the familiarity condition (*M* = 0.97 µV, SE = 0.54). Moreover, the Task condition main effect was significant at Fz, *F*(1, 92) = 6.02, *p* < .05, *η_p_^2^* = 0.06, with the mean P3 amplitude being more negative in the trustworthiness condition (*M* = −2.00 µV, SE = 0.59) compared to the familiarity condition (*M* = −0.90 µV, SE = 0.56). Thus, the mean P3 amplitude in the trustworthiness condition reveals mental effort effects, whereas the mean P3 amplitude in the familiarity condition suggests recognition of salient stimuli. Moreover, the Picture type × SI-perpetrator interaction was significant for the mean P3 amplitude, *F*(2, 184) = 5.38, *p* < .01, ε = .99, *η_p_^2^* = 0.06. To elucidate this interaction, we inserted the probe versus irrelevant P3 amplitudes and the untrustworthiness-probe versus trustworthiness P3 amplitudes in the repeated-measures analysis with the other factors being the same as in the former analysis because these two picture types required the same response (Table 2). Thus, variations of the P3 amplitude were due to the cognitive process prior to response. The Picture type × SI-perpetrator interaction was significant for both picture types, *F*(1, 92) = 8.56, *p* < .01, *η_p_^2^* = 0.09. The Pearson correlation for the mean P3 difference for untrustworthy-probe minus trustworthy faces with SI-perpetrator was significant at Pz, *r*(97) = −.22, *p* < .05, two-tailed, suggesting a less positive P3 difference score for individuals with higher SI-perpetrator scores. Similarly, the Pearson correlation was significant for the familiarity condition but at Cz, *r*(97) = −.22, *p* < .05, two-tailed, suggesting also a less positive P3 difference score for individuals with higher SI-perpetrator scores. The main effect of Task sequence was not significant, *F*(1, 92) = 0.04, *p* = .85. The interactions of Task sequence with Position, Task condition, and Picture type were not significant (all *p*s > .41). All higher-order interactions involving Task sequence (Task sequence × Position × Picture Type, Task sequence × Position × Task condition, Task sequence × Picture Type × Task condition, Task sequence × Position × Picture Type × Task condition) were not significant (all *p*s > .14).

## 3. Discussion

The present study investigated individual differences of P3 effects in two experimental settings. In the familiarity condition, participants were asked to conceal knowledge to a priori learned faces and to respond truthfully to known (target) faces and unknown (irrelevant) faces. As the familiarity condition is conceptually similar to CITs in legal settings, we expected the P3 amplitude to reflect effects of the perceived stimulus salience with the probe-P3 amplitude (known stimuli with deceptive responses) being more positive than the irrelevant-P3 amplitude (unknown stimuli with truthful responses). The trustworthiness condition was exclusively based on known faces. Participants were asked to conceal their knowledge to faces, which were predefined with a socially relevant characteristic—namely trustworthiness (cf. Gordon & Platek, 2009; Todorov, Baron, Nikolass, & Oosterhof, 2008; Willis & Todorov, 2006). That is, faces predefined as untrustworthy required a deceptive response because participants indicated by their response that they would evaluate the faces as trustworthy (untrustworthiness-probe) faces. Further faces that were predefined as trustworthy and untrustworthy, respectively, required truthful responses. We expected that mental effort might account for P3 variations in the trustworthiness CIT because all faces were learned (i.e., known) prior to task performance. The only difference between untrustworthiness-probe pictures and the other two types of pictures was the required type of response (deceptive versus truthful). When people prepare deceptive responses that are contrary to their (instructed) attitude, information processing captures two types of information – stimulus processing against one’s attitude and deceptive responses. Those situations should be more cognitively demanding than situations that incorporate known faces that require responses that are compatible with (instructed) attitudes.

The main results of the present study can be summarized as follows: Our findings demonstrate the classical region effect of the P3 amplitude. A more pronounced P3 amplitude was observed at parietal (Pz) compared to central and frontal (Cz, Fz) sites (although the overall repeated-measures ANCOVA also reveals a frontal P3 effect for the task conditions). (a) As expected, the CIT for the familiarity condition revealed a larger P3 amplitude for probe stimuli compared to irrelevant stimuli, which induced the typical effect of stimulus salience during deception (hypothesis a). This result is comparable with prior legal and social CIT P3-findings (e.g., Ambach et al., 2010; Gamer & Berti, 2010; Leue & Beauducel, 2015; Leue et al., 2012) and suggests that processing of known probe pictures is more salient than processing of unknown pictures. (b) In the trustworthiness CIT condition, we found – as expected in hypothesis (b) – smaller P3 amplitudes following untrustworthy-probe stimuli compared to trustworthy stimuli. Moreover, the P3 amplitude was marginally smaller to untrustworthy-probe stimuli compared to untrustworthy stimuli. Following the mental effort hypothesis (Beauducel et al., 2006; Kok, 2001), these results suggest that especially concealing untrustworthiness of known pictures costs more mental effort than truthful responses to known trustworthy pictures. (c) We did not find the expected interaction effect of trait-BIS and SI-perpetrator with the P3 amplitudes in the familiarity condition. In accordance with hypothesis (d), we observed that the difference between untrustworthy-probe P3 and untrustworthy P3 correlates significantly negative with SI-perpetrator, but there was no significant correlation between the difference of untrustworthy-probe P3 and trustworthy-P3. This means that concealing untrustworthiness costs more mental effort in individuals with a more pronounced perpetrator’s sensitivity to injustice when compared to truthful indicated untrustworthiness. However, we recommend to interpret this finding with caution because the Picture type × SI-perpetrator interaction of the parietal P3 amplitude just showed a tendency and the correlation between the difference of untrustworthy-probe P3 and untrustworthy P3 with SI-perpetrator is not only related to the cognitive process prior to response (because the response button differs for untrustworthy-probe and untrustworthy pictures). Therefore, the overall analysis is important showing that the P3 amplitude differences were more negative for the familiarity condition and more negative for the trustworthiness condition for individuals with higher SI-perpetrator scores, indicating that individuals with higher SI-perpetrator scores revealed a less positive probe/untrustworthiness-probe versus irrelevant/trustworthy-P3. These findings suggest that individuals with higher SI-perpetrator scores show more intense mental effort effects across both CIT-like task conditions. The rather small size of the correlations of SI-perpetrator with the P3 amplitude differences between the trustworthiness conditions indicates that several processes overlap in CIT performance and that only some of these processes are related to sensitivity to justice. The fact that we did not observe trait-BIS effects in the familiarity CIT (Leue & Beauducel, 2015; Leue et al., 2012) might be due to the fact that trait-BIS effects were overruled by SI-perpetrator effects in the extended CIT including the familiarity and the trustworthiness condition. Individual differences of SI-perpetrator modulated the P3 effects especially in the overall analysis suggesting that socially related trait dimensions like sensitivity to injustice should be taken into account when knowledge of socially salient information like (un)trustworthiness is concealed.

### 3.1 Stimulus salience or mental effort?

Our data reveal that experimental conditions differentiate between salience effects and mental effort effects of the P3 amplitude. When a social CIT captures the differentiation between known, deceptive versus unknown, truthful stimuli the P3 amplitude mirrors effects of perceived stimulus salience. When an experimental condition captures the differentiation between known stimuli with concealed versus truthfully reported untrustworthiness the P3 amplitude reflects mental effort effects. These two effects can be accounted for by the mental workload framework of the P3 (Horat et al., 2016; Mecklinger et al., 1992): When the P3 amplitude for known, deceptive stimuli has been compared with the P3 amplitude for unknown, truthful stimuli, the known stimuli attract more resources for stimulus evaluation than the unknown stimuli, leading to larger amplitudes, even when the knowledge is concealed. In contrast, when the P3 amplitude is compared for known stimuli with truthfully admitted or concealed stimulus attributes, concealing the stimulus attributes acts like a secondary task that detracts resources from stimulus evaluation and thereby reduces the P3 amplitude. Thereby, our study supports Johnson’s (2014) idea that deceptive responses can be considered in a dual-task framework. Our findings also highlight that the early P3 amplitude captures fundamental cognitive processes of stimulus evaluation but with different patterns of P3 amplitudes depending on specific social settings (i.e., concealing knowledge to faces versus concealing a social attribute like untrustworthiness). Possibly, by instruction even neutral faces can be associated with affective state attributions like trustworthiness and behavioral adaptation (Zebrowitz & Montepare, 2008). Yang et al. (2011) report that the evaluation of untrustworthiness resulted in a larger late positivity component that has been conceptually related to motivated attention. In contrast to our findings, participants were not instructed to conceal untrustworthiness. That is why our data suggest that instructed concealment of untrustworthiness demonstrates the pattern of a mentally costly event (smaller parietal P3 amplitude) instead of motivating attention and salience (leading to a larger late positivity component). This implies that the P3 amplitude difference in the familiarity condition reflects other processes than concealing information in the trustworthiness condition. In the familiarity condition the P3 amplitude difference between known probe stimuli and unknown irrelevant stimuli reflects the recognition of stimulus salience, whereas the P3 amplitude difference in the trustworthiness condition reflects the recognition of stimulus salience and a superimposed second process of suppressing knowledge of probe stimuli reflecting a mentally costly event leading to a smaller P3 amplitude difference. Future research might further elucidate the cognitive and affective processes underlying trustworthiness evaluations (Todorov et al., 2008; Willis & Todorov, 2006) and their modulation by means of individual differences (Bonnefon, Hopfensit, & De Neys, 2013; Gordon & Platek, 2009). The additional resources needed for deceptive responses according to a dual-task framework could also be related to the inhibition of arousal that has been emphasized in recent studies (klein Selle et al., 2015; Rosenfeld et al., 2017). It remains to be investigated in future studies whether the additional cognitive resources needed for deceptive responses are also related to the inhibition of arousal. Moreover, the fact that the early mean P3 amplitude was more negative at Fz for the trustworthiness condition compared to the familiarity condition (indeed) adds on the assumption that control processes (e.g., response slowing) occurred in conjunction with mental effort processes. The assumption on response times slowing in the trustworthiness condition is highlighted by the Picture type x SI-perpetrator interaction. The fact that the P3 amplitude just showed a frontality effect for the trustworthiness condition with a 4:4:4 ratio of each of the three picture types and not for the familiarity condition reveals that the 1:1:4 ratio of probe, targets versus irrelevants is unlikely to be due to a novelty effect. Moreover, the early P3 amplitude induced by the irrelevant stimuli is not larger than the P3 amplitude of the probe and target stimuli, which were known stimuli. As the early P3 amplitude of the widely unknown irrelevant stimuli was not larger than the early P3 amplitude of the known stimuli, it can be excluded that this P3 amplitude primarily captures effects of stimulus novelty. Finally, since all stimuli were faces with a neutral expression, it is unlikely that the presentation of the irrelevant, unknown, neutral faces induced a substantial amount of surprise.

### 3.2 Limitations and future directions

We used emotional neutral faces of the Radboud data set that were defined as trustworthy or untrustworthy prior to learning phase and task performance. Therefore, our P3 findings do not capture effects of the individual trustworthiness evaluations of our participants. Thus, future P3 research might investigate perceived stimulus salience and mental effort effects of trustworthy versus untrustworthy faces that were selected on the basis of consensus judgment and personal trustworthiness evaluations (e.g., Lischke, Junge, Hamm, & Weymar, 2018; Rudoy & Paller, 2009). Another opportunity to manipulate social attributes like (un)trustworthiness of faces might be given with FaceGen Software (Oosterhof & Todorov, 2008), which might help to create morphed faces that reflect (un)trustworthiness because prominent parts of the faces are affectively related to trustworthiness or untrustworthiness. Future research might also elucidate how experimental conditions and instructions (e.g., defining relations between face characteristics and social attributes) as well as contexts modulate ERP waveforms in paradigms that ask participants to deceive social attributes. In this respect, Marzi, Righi, Ottonello, Cincotta, and Viggiano (2014) reported evidence for an earlier ERP (e.g., P100) that differentiates untrustworthy versus trustworthy faces beyond the P3 amplitude. In line with Leue and Beauducel (2015), future research should also further elucidate the role of gender effects for ethically salient information processing (Dalton & Ortegren, 2011; Donoho et al., 2012). Finally, further experimental variations of deception in social settings remain to be investigated because CIT in social settings often apply a complete set of known stimuli of which some known stimuli should be concealed to and others should be truthfully responded to. Those studies often reported evidence for recognition of stimulus salience (Hu, Wu, & Fu, 2011). However, our data for the trustworthiness condition reveal evidence for the mental effort hypothesis. That is, a task setting with completely known stimuli that asks participants to conceal knowledge of a social attribute like trustworthiness adds a second process (e.g., suppression of socially relevant knowledge) that costs mental effort. The present study used the BIS/BAS scales (Strobel et al., 2001) to measure trait-anxiety. More recent psychometric studies disentangle the Fight-Flight-Freezing System (FFFS) and the BIS system more explicitly (Pugnaghi, Cooper, Ettinger, & Corr, 2017). As we did not observe the predicted trait-BIS differences, we aim at testing in a future study whether individual differences of the Carver-White BIS scale and of the BIS scale of the Corr and Cooper (2016) questionnaire (Pugnaghi et al., 2017) are related to variations of the P3 amplitude following concealed versus truthful information.

## Supplementary material

For supplementary material accompanying this paper visit http://dx.doi.org/10.1017/pen.2019.4. click here to view supplementary material
