# Supplementary material for: Does concealing familiarity evoke other processes than concealing untrustworthiness? – Different forms of concealed information modulate P3 effects
Source: Personal Neurosci. 2019 Jul 23;2:e2. doi: 10.1017/pen.2019.4 (PMC7219692; doi:10.1017/pen.2019.4)
Supplement: Supplementary file 1 [file S251398861900004Xsup001.docx]

**Supplementary material**

**Supplement S1: Radboud faces**

*Details on applied Radboud faces*

The pictures for each of the three conditions were taken from the Radboud Faces Database (RaFD, Langner et al., 2010). A total of 39 pictures showing either a face of a woman or a face of a man in frontal perspective with emotional neutral expression (Supplement S1). Averaged intensity, authenticity and valence have been calculated based on the RaFD support material (Langner et al., 2010) for each picture type. The pictures were randomly assigned to the categories of the study (familiar versus unfamiliar and trustworthy versus untrustworthy). The probability of female and male faces was equal for each of the three picture types in the familiarity CIT. Means of the intensity dimension were comparable across picture types in the familiarity condition, *F*(2, 24) < 1, *p* = .73 (familiar-probe: *M* = 3.41, *SD* = 0.28; target: *M* = 3.56, *SD* = 0.19; irrelevant (i.e., unfamiliar): *M* = 3.51, *SD* = 0.28). The same was true for the trustworthiness condition, *F*(2, 9) < 1, *p* = .72 (untrustworthy-probe: *M* = 3.60, *SD* = 0.56; trustworthy: *M* = 3.72, *SD* = 0.11; untrustworthy: *M* = 3.52, *SD* = 0.11). Means of the authenticity dimension were also comparable in the familiarity condition, *F*(2, 24) < 1, *p* = .49 (familiar-probe: *M* = 4.05, *SD* = 0.14; target: *M* = 3.82, *SD* = 0.24; irrelevant (unfamiliar): *M* = 3.98, *SD* = 0.28) and in the trustworthiness condition, *F*(2, 9) = 1.51, *p* = .86 (untrustworthy-probe: *M* = 3.99, *SD* = 0.33; trustworthy: *M* = 4.09, *SD* = 0.28; untrustworthy: *M* = 4.08, *SD* = 0.16). Finally, the means of the valence dimension were also comparable across picture types in the familiarity condition, *F*(2, 24) < 1, *p* = .97 (familiar-probe: *M* = 3.11, *SD* = 0.13; target: *M* = 3.13, *SD* = 0.28; irrelevant: *M* = 3.09, *SD* = 0.33) and in the trustworthiness condition, *F*(2, 9) = 1.51, *p* = .27 (untrustworthy-probe: *M* = 3.00, *SD* = 0.31; trustworthy: *M* = 3.28, *SD* = 0.08; untrustworthy: *M* = 3.16, *SD* = 0.22).

| Means of intensity, authenticity and valence of the applied pictures based on the RaFD support material ([Langner et al., 2010](#_ENREF_33)) | | | | | |
| --- | --- | --- | --- | --- | --- |
| RaFD-Coding | Picture type |  | Intensity | Authenticity | Valence |
|  |  |  | ***M*** | ***M*** | ***M*** |
| Female faces |  |  |  |  |  |
| Rafd090_01_Caucasian_female_neutral_frontal | familiar-probe |  | 3.48 | 4.04 | 3.04 |
| Rafd090_02_Caucasian_female_neutral_frontal | familiar-probe |  | 3.61 | 4.22 | 3.04 |
| Rafd090_04_Caucasian_female_neutral_frontal | target |  | 3.70 | 3.78 | 3.17 |
| Rafd090_08_Caucasian_female_neutral_frontal | target |  | 3.52 | 3.68 | 2.96 |
| Rafd090_14_Caucasian_female_neutral_frontal | irrelevant |  | 3.54 | 3.92 | 2.85 |
| Rafd090_18_Caucasian_female_neutral_frontal | irrelevant |  | 3.27 | 3.68 | 2.95 |
| Rafd090_19_Caucasian_female_neutral_frontal | untrustworthy-probe |  | 3.62 | 3.96 | 2.92 |
| Rafd090_22_Caucasian_female_neutral_frontal | untrustworthy-probe |  | 3.88 | 4.29 | 2.96 |
| Rafd090_26_Caucasian_female_neutral_frontal | untrustworthy |  | 3.58 | 3.67 | 3.25 |
| Rafd090_27_Caucasian_female_neutral_frontal | untrustworthy |  | 3.70 | 4.17 | 3.22 |
| Rafd090_31_Caucasian_female_neutral_frontal | trustworthy |  | 3.61 | 4.22 | 3.35 |
| Rafd090_32_Caucasian_female_neutral_frontal | trustworthy |  | 3.58 | 3.96 | 3.08 |
| Rafd090_37_Caucasian_female_neutral_frontal | irrelevant |  | 3.54 | 4.17 | 3.46 |
| Rafd090_56_Caucasian_female_neutral_frontal | irrelevant |  | 3.83 | 4.21 | 3.46 |
| Rafd090_57_Caucasian_female_neutral_frontal | irrelevant |  | 3.46 | 3.63 | 3.21 |
| Rafd090_58_Caucasian_female_neutral_frontal | irrelevant |  | 3.36 | 4.23 | 3.05 |
| Rafd090_61_Caucasian_female_neutral_frontal | irrelevant |  | 3.52 | 4.35 | 3.17 |
| Rafd090_16_Caucasian_female_neutral_frontal | irrelevant |  | 3.14 | 3.38 | 2.52 |
| Rafd090_12_Caucasian_female_neutral_frontal | irrelevant |  | 4.04 | 4.17 | 3.48 |
| Male faces |  |  |  |  |  |
| Rafd090_03_Caucasian_male_neutral_frontal | familiar-probe |  | 3.00 | 3.87 | 3.30 |
| Rafd090_05_Caucasian_male_neutral_frontal | familiar-probe |  | 3.55 | 4.05 | 3.05 |
| Rafd090_07_Caucasian_male_neutral_frontal | target |  | 3.71 | 4.17 | 3.50 |
| Rafd090_09_Caucasian_male_neutral_frontal | target |  | 3.30 | 3.65 | 2.87 |
| Rafd090_10_Caucasian_male_neutral_frontal | irrelevant |  | 3.55 | 3.82 | 2.95 |
| Rafd090_15_Caucasian_male_neutral_frontal | irrelevant |  | 3.62 | 4.14 | 3.33 |
| Rafd090_20_Caucasian_male_neutral_frontal | untrustworthy-probe |  | 2.82 | 3.55 | 2.68 |
| Rafd090_23_Caucasian_male_neutral_frontal | untrustworthy-probe |  | 4.09 | 4.17 | 3.43 |
| Rafd090_24_Caucasian_male_neutral_frontal | untrustworthy |  | 3.84 | 4.24 | 3.24 |
| Rafd090_25_Caucasian_male_neutral_frontal | untrustworthy |  | 3.74 | 4.26 | 3.39 |
| Rafd090_28_Caucasian_male_neutral_frontal | trustworthy |  | 3.36 | 3.92 | 2.88 |
| Rafd090_30_Caucasian_male_neutral_frontal | trustworthy |  | 3.54 | 4.21 | 3.33 |
| Rafd090_33_Caucasian_male_neutral_frontal | irrelevant |  | 3.92 | 3.96 | 3.21 |
| Rafd090_36_Caucasian_male_neutral_frontal | irrelevant |  | 3.92 | 4.04 | 3.24 |
| Rafd090_38_Caucasian_male_neutral_frontal | irrelevant |  | 3.43 | 4.19 | 2.81 |
| Rafd090_46_Caucasian_male_neutral_frontal | irrelevant |  | 3.63 | 4.33 | 2.96 |
| Rafd090_47_Caucasian_male_neutral_frontal | irrelevant |  | 3.00 | 3.59 | 3.05 |
| Rafd090_49_Caucasian_male_neutral_frontal | irrelevant |  | 3.15 | 3.92 | 3.19 |
| Rafd090_71_Caucasian_male_neutral_frontal | irrelevant |  | 3.55 | 4.20 | 3.50 |
| Rafd090_21_Caucasian_male_neutral_frontal | irrelevant |  | 3.23 | 3.69 | 2.27 |
|  | | | | | |

**Supplement S2 | Instructions**

**
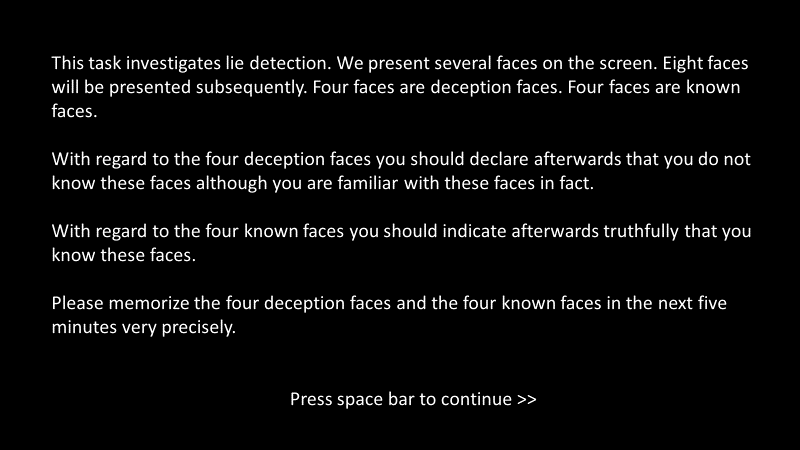
**

**
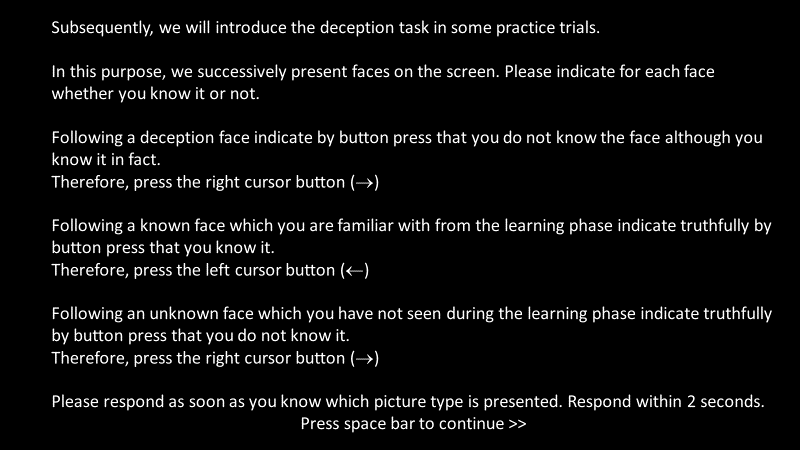
**

**
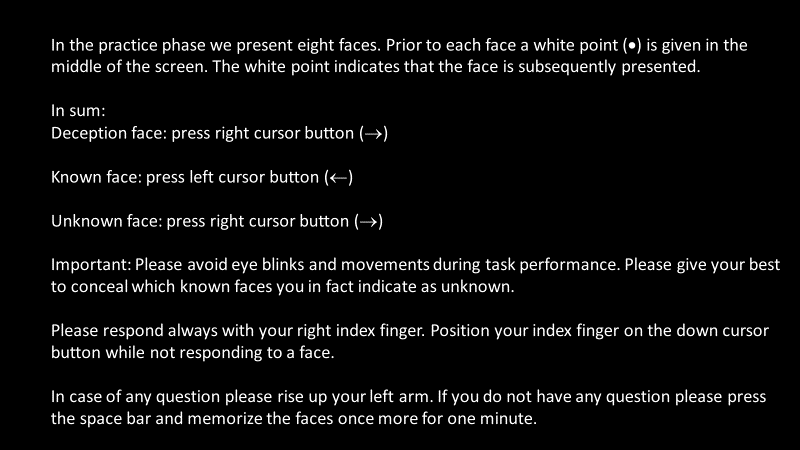
**

**
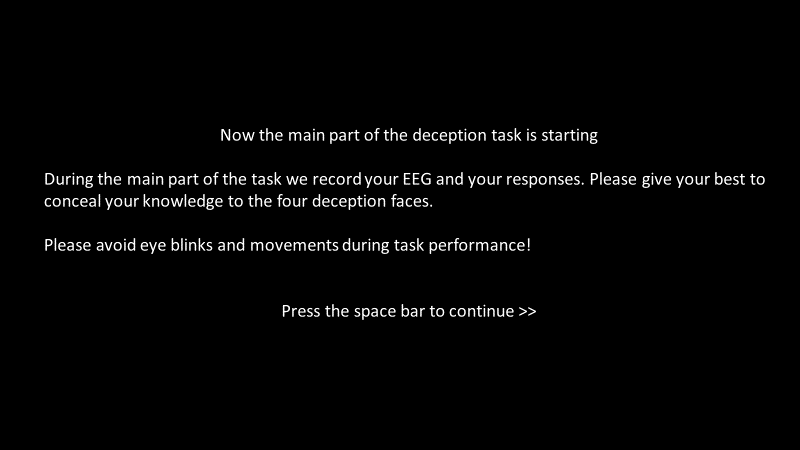
**

**
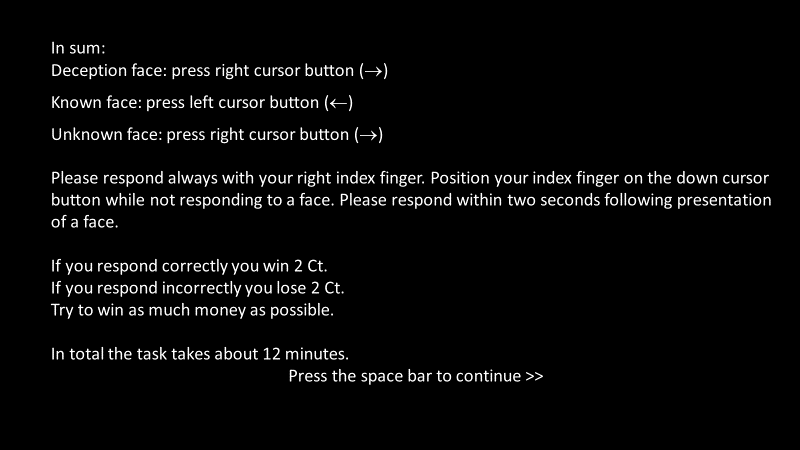
**

**
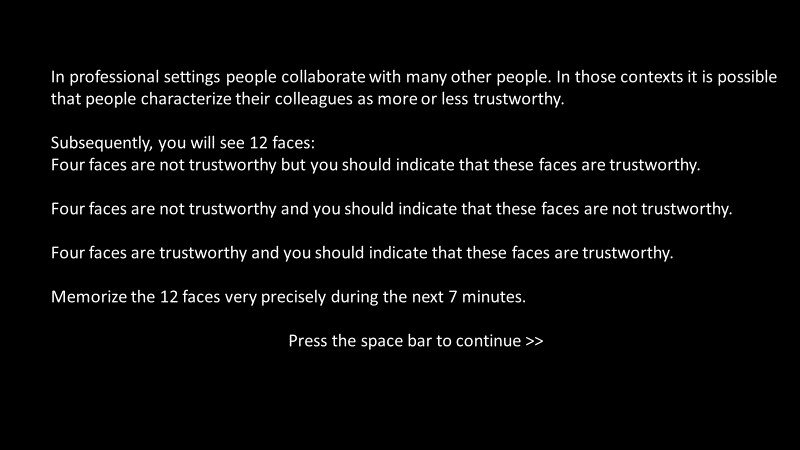
**

**
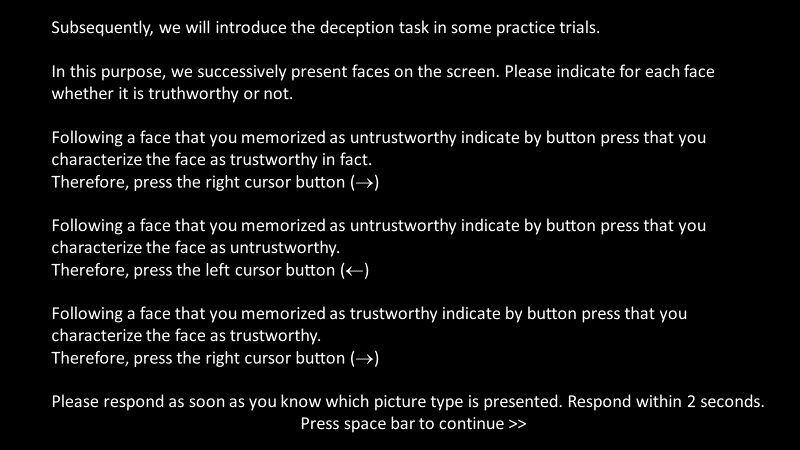
**

**
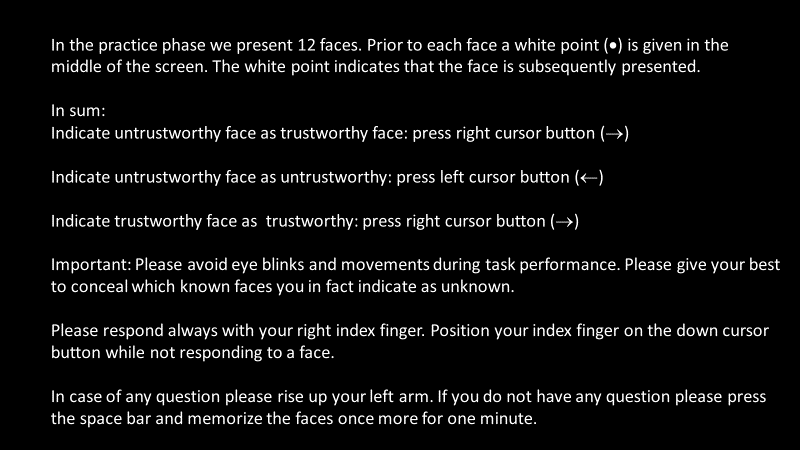
**

**
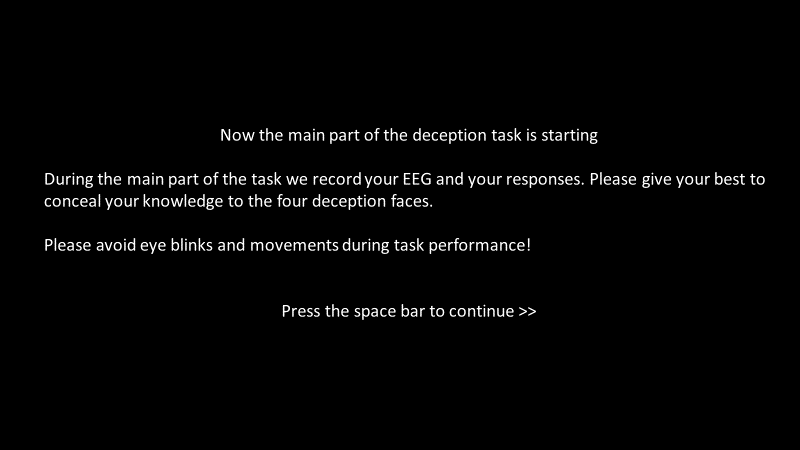
**

**
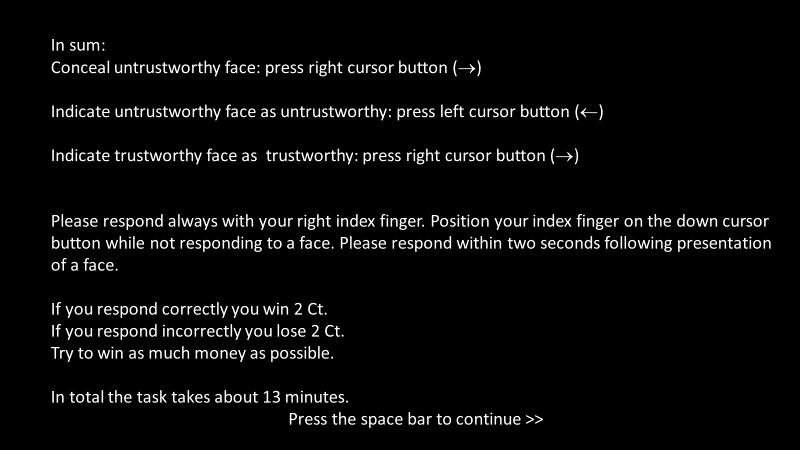
**

**Supplement S3 | Mean P3b quantification & results**

Grand averages of the picture-related ERPs (0-1000 ms, with 100 ms pre-stimulus-baseline) indicate a late P3 amplitude between 450 and 680 ms post-stimulus (Figure 2). The second P3 component (P3b) was quantified as a mean amplitude in the time interval between 450 to 680 ms post-stimulus. As the hypotheses were tested for a second time with this dependent variable, we used a nominal alpha level of *p* < .025 for significance testing.

*Mean P3b amplitude of the familiarity condition*

For the P3b amplitude, there was a main effect of Position, *F*(2, 186) = 153.93, *ε* = .65, *p* < .01, *ƞ_p_^2^* = 0.62. Simple contrasts revealed the mean P3b amplitude was larger at Pz compared to Cz, *F*(1, 93) = 101.29, *p* < .01, *ƞ_p_^2^* = 0.521, and it was larger at Pz compared to Fz, *F*(1, 93) = 178.24, *p* < .01, *ƞ_p_*^2^ = 0.66 (Table S2). Since the Position main effect indicated the typical parietal P3 topography, further analyses have been conducted at Pz. At Pz, the Picture type main effect was significant for the P3b amplitude, *F*(2, 186) = 4.31, *ε* = .92, *p* < .025, *ƞ_p_^2^* = 0.04. Simple contrasts revealed that the P3b amplitude did not significantly differ between familiar-probe and irrelevant stimuli, *F*(1, 93) = 0.18, *n.s.*. The P3b amplitude was significantly higher for target stimuli than for familiar-probe stimuli, *F*(1, 93) = 10.70, *p* < .01., *ƞ_p_^2^* = 0.10. There was a significant main effect of Sex, *F*(1, 93) = 4.34, *p* < .025, *ƞ_p_^2^* = 0.05 with higher P3b amplitudes for females (*M* = 9.44 µV, *SE* = 0.78 µV) compared to males (*M* = 7.01 µV, *SE* = 0.77 µV). We did not find main effects of Trait-BIS and SI-perpetrator, (all *p*s > .69). We also did not observe interactions of Picture type × Sex, Picture type × Trait-BIS and Picture type × SI-perpetrator for the mean Pb3 amplitude (all *p*s > .18).

---- Please insert Table S2 about here ----

*Mean P3b amplitude of the trustworthiness condition*

The main effect of Position was significant, *F*(2, 186) = 159.84, *ε* = .64, *p* < .01, *ƞ_p_^2^* = 0.63. Simple contrasts revealed that the mean P3b amplitude was larger at Pz compared to Cz, *F*(1, 93) = 104.11, *p* < .01, *ƞ_p_^2^* = 0.53, and it was larger at Pz compared to Fz, *F*(1, 93) = 184.86, *p* < .01, *ƞ_p_*^2^ = 0.67 (Table S2). Since the Position main effect indicated the typical parietal P3 topography, further analyses have been conducted for the P3b at Pz. At Pz, the Picture type main effect was significant, *F*(2, 186) = 13.47, *ε* = .98, *p* < .01, *ƞ_p_^2^* = 0.13. Simple contrasts revealed the P3b amplitude was marginally significant smaller for untrustworthiness-probe than for trustworthy stimuli, *F*(1, 93) = 3.51, *p* = .06, *ƞ_p_^2^* = 0.04 (Table S2), and significantly smaller for untrustworthiness-probe stimuli than for untrustworthy stimuli, *F*(1, 93) = 23.60, *p* < .01, *ƞ_p_^2^* = 0.04. There was a significant main effect of Sex, *F*(1, 93) = 7.49, *p* < .01, *ƞ_p_^2^* = 0.08 , with higher P3b amplitudes for females (*M* = 9.73 µV, *SE* = 0.65 µV) compared to males (*M* = 7.14 µV, *SE* = 0.64 µV). There were no main effects of Trait-BIS or SI-perpetrator (all *p*s > .46), and no interaction of Picture type × Sex, Picture type × Trait-BIS or Picture type × SI-perpetrator (all *p*s > .25).

**Table S3 |** Mean P3b amplitudes (in µV).

| **Familiarity** |  |  |  |  |  | **Trustworthiness** |  |  |  |  |
| --- | --- | --- | --- | --- | --- | --- | --- | --- | --- | --- |
| *electrode position* |  | P3 amplitudes | | |  | *electrode position* |  | P3 amplitudes | | |
| Pz |  | 8.25 | (0.53) |  |  | Pz |  | 8.44 | (0.44) |  |
| Cz |  | 4.28 | (0.48) |  |  | Cz |  | 3.93 | (0.51) |  |
| Fz |  | -0.12 | (0.49) |  |  | Fz |  | -0.42 | (0.59) |  |
| *picture type^1^* |  |  |  |  |  | *picture type^1^* |  |  |  |  |
| familiar-probe |  | 8.02 | (0.54) |  |  | untrustworthy-probe |  | 7.93 | (0.44) |  |
| Target |  | 8.62 | (0.56) |  |  | Trustworthy |  | 8.32 | (0.46) |  |
| Irrelevant |  | 8.12 | (0.53) |  |  | Untrustworthy |  | 9.06 | (0.48) |  |
| *Note.* Standard error of mean is given in parentheses. ^1^Mean P3b amplitudes for each picture type at Pz. | | | | | | | | | | |

**Supplement S4 | temporal principal component analysis (PCA) of ERP data**

To investigate whether the early and late P3 can be separated and to give a more detailed account of the ERP components, we performed a temporal PCA of the ERP data at Pz for the probes of the familiarity condition and for the probes of the trustworthiness condition. We used covariance-based PCA with subsequent Promax-rotation ([Kayser and Tenke, 2003](#_ENREF_28);[Dien, 2010](#_ENREF_10)).

Assuming that components explaining less than 1% of the variance represent noise, only components that explain with at least 1% of explained variance were retained for Promax-rotation. On this basis, seven components explaining 94% of the total variance were retained for rotation in the familiarity condition. There is a rather large late P3 with a loading peak at about 700 ms (gray line, Figure S4A). However, there is also a quite large early P3 with a loading peak at about 300 ms (bold line, Figure S4A). The Pearson correlation between these two components is *r* = .57, *p* < .01, two-tailed, indicating that in spite of some amount of common variance they represent different aspects of the P3.

In the temporal PCA for the trustworthiness paradigm, there were eight components each single component explaining at least 1% of the variance. These eight components explain about 94% of the total variance. The rotated loadings of the temporal PCA of the trustworthiness paradigm indicate a rather late component (tPC800) with a loading peak at about 800 ms, a second component (tPC500) with a loading peak at about 500 ms (dotted line, Figure S4B), and an early component (tPC270) with a loading peak at about 270 ms (bold line, Figure S4B).

(A)

tPC 500

tPC 270 tPC 800

(B)

tPC 270 tPC 500 tPC 800

**Figure S4** **|** Promax-rotated (Kappa=4) component-loadings for the familiarity condition (A) and for the trustworthiness condition (B).

Thus, there were three components with a loading peak between 270 and 800 ms. The Pearson correlation between the earliest with the latest component in the time range between 270 and 800 ms was *r* = .61, *p* < .01, two-tailed, the correlation of the earliest component with the second component was *r* = .31, *p* < .01, two-tailed, and the correlation of the second component (tPC500) with the last component (tPC800) is *r* = .49, *p* < .01, two-tailed. Again, the correlations were substantial but they do not indicate that the components should be collapsed. Overall, the temporal PCAs reveal that a single time-window for a late P3 component would not represent the complexity of the data at hand as in both analyses a substantial early P3 occurred.
